# Medicinal attributes of major phenylpropanoids present in cinnamon

**DOI:** 10.1186/s12906-016-1147-4

**Published:** 2016-05-31

**Authors:** Uma Kant Sharma, Amit Kumar Sharma, Abhay K. Pandey

**Affiliations:** Department of Biochemistry, University of Allahabad, Allahabad, 211002 India

**Keywords:** Cinnamaldehyde, Eugenol, Phenylpropanoid, Free radical, Antioxidant, Antibacterial, Antiproliferative, Lipoprotective

## Abstract

**Background:**

Excessive production of free radicals has been implicated in many diseases including cancer. They are highly reactive and bring about oxidation of biomolecules i.e., proteins, lipids and nucleic acids which are associated with many degenerative diseases. Natural products acting as antioxidants have ability to neutralize free radicals and their actions and hence they mitigate their harmful effects. The present study was designed to investigate pharmacological properties viz., antioxidant, antibacterial and antiproliferative activities of cinnamaldehyde and eugenol, the two naturally occurring phenylpropanoids present in *Cinnamomum* spp. and other plants.

**Methods:**

The antioxidant potential of test compounds was evaluated by measuring DPPH free radical scavenging, reducing power and metal ion chelating activities. Protection against membrane damage was assayed by inhibition of lipid peroxidation in rat liver homogenate. Antibacterial activity was measured by Kirby-Bauer disc diffusion method while antiproliferative activity of test compounds was measured by sulforhodamine-B (SRB) assay.

**Results:**

Eugenol exhibited noticeable antioxidant potential in DPPH radical scavenging (81 %) and reducing power (1.12) assays at 1.0 μM/ml and 0.1 μM/ml concentrations, respectively. IC_50_ value of eugenol for radical scavenging activity was found to be 0.495 μM/ml. Cinnamaldehyde demonstrated considerable metal ion chelating ability (75 %) at 50 μM/ml and moderate lipo-protective activity in lipid peroxidation assay at 3 μM/ml. In addition cinnamaldehyde also showed appreciable antibacterial activity (zone of inhibition 32–42 mm) against *Bacillus cereus* (MTCC 6840), *Streptococcus mutans* (MTCC 497), *Proteus vulgaris* (MTCC 7299), *Salmonella typhi* (MTCC 3917) and *Bordetella bronchiseptica* (MTCC 6838) while eugenol produced moderate activity at 80 μM/disc. Cinnamaldehyde exhibited comparatively better antiproliferative potential against breast (T47D) and lung (NCI-H322) cancer cell lines than eugenol in SRB assay at 50 μM concentration.

**Conclusion:**

Cinnamaldehyde possessed metal ion chelating, lipo-protective, antibacterial and antiproliferative activities while eugenol showed potent H-atom donating potential indicating radical quenching and reducing power abilities. Medicinal attributes shown by both the compounds indicated their usefulness in food and pharmaceutical sector.

## Background

Free radicals are associated with many degenerative diseases including cancer, cardio-vascular diseases, cataract, immune system decline and brain dysfunction [1]. Under normal metabolic conditions about 2–5 % of O_2_ consumed by mitochondria is converted to ROS (Reactive oxygen species) during metabolic process within the body [2]. Their excessive production during abnormal conditions is regulated naturally by antioxidant system [3]. Failure of antioxidant defenses results in a pathophysiological condition known as oxidative stress. Highly active oxygen and nitrogen species (ROS and RNS) are generally considered to be markers of oxidative stress. They permanently modify the genetic material leading to numerous degenerative or chronic diseases [4]. Mis-repair of DNA damage results in mutations such as base substitution and deletion which lead to carcinogenesis [5].

Antioxidants are a group of substances which are either produced in situ or supplied through food and supplements. They protect free radical mediated membrane damage because of their scavenging and chelating properties [6, 7]. Butylated hydroxyanisole (BHA) and butylated hydroxytoluene (BHT) are commonly used as synthetic antioxidant but their uses are restricted by legislative rules because of doubts over their toxic and carcinogenic effects [8]. Antioxidants derived from plants are presumed to be safe since they are natural in origin and have capability to counteract the damaging effect of ROS [9].

Many microorganisms cause food spoilage which is one of the most important concerns of the food industry [10]. Initially, synthetic chemicals were used to prevent microbial contamination as well as oxidation of dietary components so that they remained in their natural form. Because of the growing concern of consumer about the side effects of synthetic compounds and want of safer material for preventing and controlling pathogenic microorganisms in food, natural products are currently being used to prevent microbial contamination [11]. Phytochemicals have been reported to modulate human metabolism in a manner beneficial for the prevention of infectious and degenerative diseases [12, 13].

**Fig. 1:**
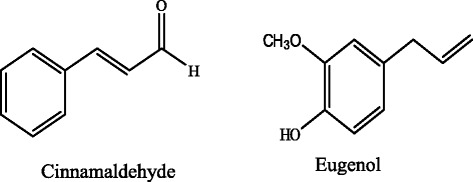
Chemical structures of cinnamaldehyde (3-Phenyl-2-propenal) and eugenol (2-Methoxy-4-(2-propenyl) phenol)

Spices and aromatic vegetable material of natural origin are used in food industries to enhance flavor and fragrance qualities of food materials. Moreover they are also used as traditional medicine [14]. Spices are good sources of natural antioxidant and antibacterial agents. Cinnamon and bay leaf are used as spices and obtained from *Cinnamomum* spp. Principle components like cinnamaldehyde, eugenol, cinnamic acid and cineol etc. are responsible for the antioxidant activity in cinnamon [15]. Very low amount of eugenol is usually present in cinnamon bark but it is the major component of cinnamon leaf essential oil. It is also abundantly present in *Syzygium aromaticum* (clove). Both cinnamaldehyde and eugenol belong to phenylpropanoid class of phytochemicals. Cinnamaldehyde bears an aldehyde group on benzene ring via three carbon chain while eugenol has one hydroxy and one methoxy group which are directly attached to the ring (Fig. 1). Eugenol has a wide range of application in perfumes, flavorings, essential oils and in medicine as a local antiseptic and anesthetic [16]. Present study reports antioxidant, antibacterial and antiproliferative activities of cinnamaldehyde and eugenol, the two flavoring phenylpropanoid compounds.

## Methods

### Chemicals

Cinnamaldehyde (3-phenyl-2-propenal), eugenol (2-methoxy-4-(2-propenyl) phenol), DPPH (2, 2-diphenyl-1-picryl hydrazyl), tert-butyl-4-hydroxytoluene (BHT), butylated hydroxyanisole (BHA) were obtained from Himedia, Pvt. Ltd Mumbai, India.

### Assessment of antioxidant ability by in vitro assays

#### Free radical (DPPH) scavenging assay

The hydrogen-donating ability of cinnamaldehyde and eugenol was examined in the presence of DPPH, a stable radical using the method of Singh et al. [17]. One ml cinnamaldehyde (0.4–4.0 mM/ml) and eugenol (0.4–4.0 μM/ml) prepared in DMSO were taken in different test tubes and 3 ml of 0.1 mM DPPH solution prepared in methanol was added. The content was mixed and allowed to stand at room temperature for 30 min in dark. Final concentration of cinnamaldehyde and eugenol in reaction mixture was 0.1–1.0 mM/ml and 0.1–1.0 μM/ml, respectively. The reduction of DPPH free radical was measured by recording the absorbance at 517 nm. BHA was used as standard for comparison. Control tubes contained 1 ml DMSO and 3 ml DPPH reagent in reaction mixture. The radical scavenging activities (%) at different concentrations of the test samples were calculated using the following formula.

$$ \mathrm{Free}\ \mathrm{radical}\ \mathrm{scavenging}\ \mathrm{activity}\left(\%\right)=\left[\left({\mathrm{A}}_{\mathrm{C}}-{\mathrm{A}}_{\mathrm{S}}\right)/{\mathrm{A}}_{\mathrm{C}}\right]\times 100 $$

where A_C_ and A_S_ are the absorbance values of the control and the sample, respectively. IC_50_ value, the concentration of sample exhibiting 50 % free radical scavenging activity, was also determined by non-linear regression analysis using GraphPad Prism software.

### Reducing power assay

The reducing power of the sample was determined by the method of Oyaizu [18]. DMSO was used as solvent to make different concentrations of samples. 1.0 ml of sample and standard was placed in different test tubes. To each test tube 2.5 ml of phosphate buffer (0.2 M, pH 6.6) and 2.5 ml of 1 % potassium hexa-cyanoferrate (K_3_Fe(CN)_6_) were added and contents were vortexed. Final concentration of cinnamaldehyde, eugenol and ascorbic acid in reaction mixture was 0.02–0.1 μm/ml. Tubes were then incubated at 50 °C in a water bath for 20 min. The reaction was stopped by adding 2.5 ml of 10 % TCA solution and then centrifuged at 4000 rpm for 10 min. After centrifugation 1.0 ml of the supernatant was mixed with 1 ml of distilled water and 0.5 ml of ferric chloride solution (0.1 %, w/v) and kept at room temperature for 2 min. The reaction led to formation of greenish blue colour. The absorbance was measured at 700 nm and higher absorbance values denoted better reducing power of the test samples. Ascorbic acid was used as standard for comparison.

### Lipid peroxidation inhibition activity (LPOI)

Lipo-protective efficacy of samples was estimated by the method of Halliwell and Gutteridge [19]. The study was performed in accordance with the Guide for the Care and Use of Laboratory Animals, as promulgated by CPCSEA India and adopted by Institutional Animal Ethics Committee, University of Allahabad, Allahabad. The liver tissue was isolated from normal albino Wistar rats and 10 % (w/v) homogenate was prepared in phosphate buffer (0.1 M, pH 7.4 having 0.15 M KCl) using homogenizer. The homogenate was centrifuged at 800 g for 15 min and clear cell free supernatant was used for in vitro lipid peroxidation inhibition assay. 100 μl of different concentrations of samples was taken in different tubes, followed by addition of 1.0 ml KCl (0.15 M), 0.3 ml phosphate buffer and 0.5 ml of tissue homogenate. Peroxidation was initiated by adding 100 μl FeCl_3_ (0.2 mM). Final concentration of cinnamaldehyde, eugenol and BHA (standard) in reaction mixture was 1.0–3.0 μM/ml. After incubation at 37 °C for 30 min, lipid peroxidation was monitored by the formation of thiobarbituric acid reactive substances which were estimated by adding 2 ml of ice-cold hydrochloric acid (0.25 N) containing 15 % TCA, 38 % TBA and 0.5 % BHT. The reaction mixture was incubated at 80 °C for 1 h followed by cooling and centrifugation. The absorbance of the pink supernatant was measured at 532 nm. All analyses were carried out in triplicate and results were expressed as mean ± SD. The protective effect of extracts against lipid peroxidation (% LPOI) was calculated by using the following formula.

$$ \mathrm{LPOI}\left(\%\right)=\left[\left(\mathrm{A}\mathrm{c}-\mathrm{A}\mathrm{s}\right)/\mathrm{A}\mathrm{c}\right]\times 100 $$

where Ac is absorbance of control and As is absorbance in the presence of the sample or standard compounds.

### Metal ion chelating activity

The chelating activity of the cinnamaldehyde and eugenol was measured by the method of Dinis et al. [20]. Samples (200 μl) containing different concentrations were prepared in methanol followed by addition of 50 μl of FeCl_2_ (2.0 mM). The reaction was initiated by addition of 200 μl of 50 mM ferrozine and the reaction mixture was shaken vigorously and left standing at room temperature for 10 min. Concentration of cinnamaldehyde and eugenol in final reaction mixture was 5–50 μM/ml. After the mixture had reached equilibrium, the absorbance of the pink violet colour solution was measured spectrophotometrically at 562 nm by using a UV-Visible spectrophotometer (Visiscan 067). BHA and EDTA were used for comparison. The control contained FeCl_2_ and ferrozine, without samples. The percentage inhibition of ferrozine-Fe^2+^ complex formation was measured in form of metal chelating activity.

$$ \%\ \mathrm{Metal}\ \mathrm{ion}\ \mathrm{c}\mathrm{helating}\ \mathrm{activity}=\left[\left(\mathrm{A}\mathrm{c}-\mathrm{A}\mathrm{s}\right)/\mathrm{A}\mathrm{c}\right]\times 100 $$

where Ac is absorbance of control and As is absorbance in the presence of the sample or standard compounds.

### Antibacterial activity assessment

#### Bacterial strains

Gram negative [*Proteus vulgaris* (MTCC 7299)*, Salmonella typhi* (MTCC 3917) and *Bordetella bronchiseptica* (MTCC 6838)] and Gram positive [*Bacillus cereus* (MTCC 6840) and *Streptococcus mutans* (MTCC 497)] bacteria were obtained from IMTECH, Chandigarh, India.

### Disc diffusion method for antimicrobial activity assay

Antimicrobial activity of each plant extract was determined using Kirby-Bauer disc diffusion method [21]. Briefly, 100 μl of the test bacteria was inoculated in 10 ml of fresh nutrient media until they reached a count of approximately 10^8^cells/ml. From the log phase culture, 100 μl of the microbial suspension was spread onto Muller Hinton agar plates. Sterile discs (diameter 6 mm, Hi Media) were impregnated with 20 μl of the test sample, and placed onto inoculated plates followed by incubation at 37 °C for 24 h. Standard antibiotic discs (Meropenem 10 μg and vancomycin 30 μg) were used as control. Diameter of the inhibition zones were measured in millimeters (mm) and results were reported as average of three replicates.

### Evaluation of antiproliferative activity by SRB assay

The in vitro antiproliferative activity of test compounds was determined using sulforhodamine-B dye (SRB) assay [22]. Cell suspension (100 μl, 1 × 10^5^ to 2 × 10^5^ cells per ml depending upon mass doubling time of cells) was grown in 96-well tissue culture plate and incubated for 24 h. Stock solutions of test compounds were prepared in DMSO and serially diluted with growth medium to obtain desired concentrations. 100 μl samples (100 μM) were then added to the wells and cells were further incubated for another 48 h. The cell growth was arrested by layering 50 μl of 50 % TCA and incubated at 40 °C for an hour followed by washing with distilled water and then air-dried. SRB (100 μl, 0.4 % in 1 % acetic acid) was added to each well and plates were incubated at room temperature for 30 min. The unbound SRB dye was washed with 1 % acetic acid and then plates were air dried. Tris–HCl buffer (100 μl, 0.01 M, pH 10.4) was added and the absorbance was recorded on ELISA reader at 540 nm. Each test was done in triplicate. The values are reported as mean ± SD of three replicates.

### Statistical analysis

All experiments were carried out in triplicate and data were represented as mean ± standard deviation (SD). Graphs were prepared using GraphPad Prism software. Data were analyzed using One-way ANOVA and the values of $P$ <0.05 were considered as statistically significant.

## Results

### DPPH free radical scavenging assay

**Fig. 2:**
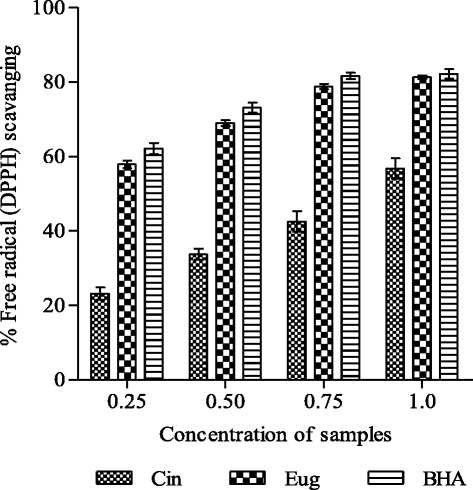
DPPH free radical scavenging ability of cinnamaldehyde (Cin) and eugenol (Eug). Results are shown as mean ± SD of three replicates (*P* <0.05). Concentrations are expressed as mM/ml (Cin) and μM/ml (Eug and BHA)

Radical scavenging potential of compounds was determined by measuring the degree of discoloration of DPPH solution. Eugenol exhibited strong antioxidant potential (58–81 %) at all test concentrations (0.25–1.0 μM/ml) while cinnamaldehyde showed lower to moderate radical scavenging ability (23–57 %) (Fig. 2). The DPPH radical scavenging potential of eugenol was comparable to the activity shown by the standard antioxidant BHA (62–82 %). IC_50_ value for eugenol and cinnamaldehyde were found to be 0.495 μM/ml and 0.842 mM/ml, respectively.

### Reducing power assay

**Fig. 3:**
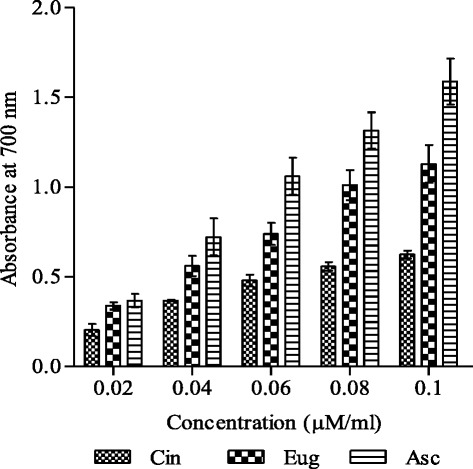
Reducing power ability of cinnamaldehyde and eugenol. Reducing power and concentration of compounds are expressed as absorbance and μM/ml, respectively. Ascorbic acid was used as standard reducing agent for comparison. Results are shown as mean ± SD (*n* = 3, *P* <0.05). Abbreviations: Cin- cinnamaldehyde, Eug-eugenol and Asc- ascorbic acid

Reducing power ability of test compounds exhibited the similar pattern as observed in radical scavenging assay. Eugenol showed appreciable reducing ability (absorbance 0.33–1.12) as compared to cinnamaldehyde (0.20–0.62) in the concentration range 0.02–0.1 μM/ml (Fig. 3). However ascorbic acid accounted for slightly higher reducing power (0.36–1.58). Cinnamaldehyde and eugenol exhibited about 55 and 92 % reducing power of ascorbic acid, respectively, at lower concentration (0.02 μM/ml) while at higher concentration (0.1 μM/ml) they showed 40 and 71 % activity of ascorbic acid, respectively.

### Lipid peroxidation inhibition activity

**Fig. 4:**
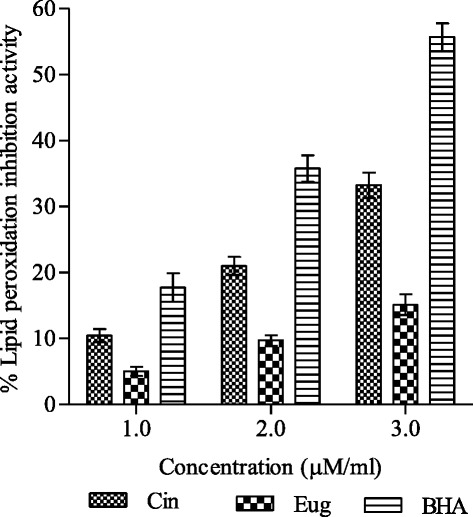
Lipoprotective efficacy of cinnamaldehyde and eugenol in rat liver homogenate. Lipid peroxidation inhibition (% LPOI) was determined at different concentrations (1.0, 2.0, 3.0 μM/ml) as described in methods. BHA was used as standard lipoprotective agent. The results are expressed as mean ± SD of three replicates (*P* <0.05). Abbreviations: *Cin* cinnamaldehyde, *Eug* eugenol, *BHA* butylated hydroxyanisole

Cinnamaldehyde and eugenol mediated protective effect on metal induced lipid peroxidation was measured in liver homogenate of albino Wistar rats in vitro and is represented as % LPOI (lipid peroxidation inhibition). Test compounds exhibited low to moderate activity. Cinnamaldehyde showed comparatively better protective action (LPOI 11–33 %) against peroxidative damage in the concentration range 1–3 μM/ml (Fig. 4). Under same test conditions eugenol accounted for lower activity (LPOI 5–15 %). BHA was used for comparison, which produced 18–56 % lipoprotective activity. Malondialdehyde produced by lipid peroxidation forms a pink chromogenic substance after reaction with thiobarbituric acid (TBA) which makes the basis for this measurement.

### Metal ion chelating activity

**Fig. 5:**
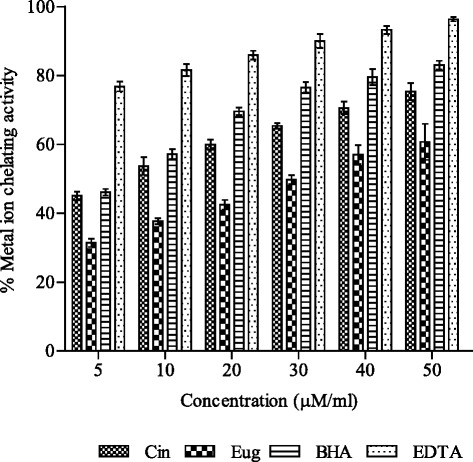
Metal ion chelating activity (%) of cinnamaldehyde and eugenol. BHA and EDTA were used for comparison. The activity of samples was represented as mean ± SD of three replicates ($P$ <0.05). Abbreviations: *Cin* cinnamaldehyde, *Eug* eugenol, *BHA* butylated hydroxyanisole and *EDTA* ethylenediaminetetraacetic acid

Cinnamaldehyde showed better chelating ability as compared to eugenol at all test concentrations (Fig. 5). BHA and EDTA were used as standard chelating agents for comparison. Cinnamaldehyde exhibited about 45–75 % metal ion chelating ability in the concentration range 5–50 μM/ml which is equivalent to 59–78 % and 90–98 % of the activities demonstrated by EDTA and BHA, respectively (Fig. 5). Similarly eugenol produced 41–60 % activity (EDTA equivalent) and 68–73 % (BHA equivalent).

### Antibacterial activity

Bacteria used in the study showed susceptibility to test samples (Table 1). Cinnamaldehyde exhibited remarkable activity against both Gram positive [*B. cereus* (MTCC 6840), *Streptococcus mutant* (MTCC 497)] and Gram negative [*P. vulgaris* (MTCC7299), *S. typhi* (MTCC 3917), *B. bronchiseptica* (MTCC 6838)] bacteria. Concentration dependent response was observed in the activity pattern. At 20 μM/disc inhibition zones produced by cinnamaldehyde ranged between 22 and 30 mm while at concentration 80 μM/disc appreciable activity was observed (inhibition zones 32–40 mm). However saturation effect was observed at further higher concentration (120 μM/disc). Eugenol in general showed lower to moderate response (inhibition zones 9–18 mm) at test concentrations. Meropenem and vancomycin exhibited 18–27 mm zone of inhibition against test bacteria. Results indicated that cinnamaldehyde has potent antibacterial activity.

**Table 1:** Antibacterial activity of cinnamaldehyde and eugenol

Test bacteria	20 μM/disc		80 μM/disc		120 μM/disc		Std. antibiotic
	Cin	Eug	Cin	Eug	Cin	Eug	
*Proteus vulgaris* (MTCC 7299)	22	10	35	13	36	15	27^a^
*Salmonella typhi* (MTCC 3917)	27	10	35	13	37	16	25^a^
*Bacillus cereus* (MTCC 6840)	30	12	40	15	42	18	24^b^
*Streptococcus mutans* (MTCC 497)	30	9	32	12	36	16	18^b^
*Bordetella bronchiseptica* (MTCC 6838)	26	10	38	14	40	18	22^b^

Antibacterial activity was measured at three different concentrations (20, 80 and 120 μM/disc) as described in methods section. Inhibition zones (mm) are reported as average of three replicates. Antibiotics were used as positive control. Abbreviations: *Cin* cinnamaldehyde, *Eug* eugenol, *std.* standard, *a* meropenem (10 μg/disc), *b* vancomycin (30 μg/disc)

### Evaluation of antiproliferative activity

**Fig. 6:**
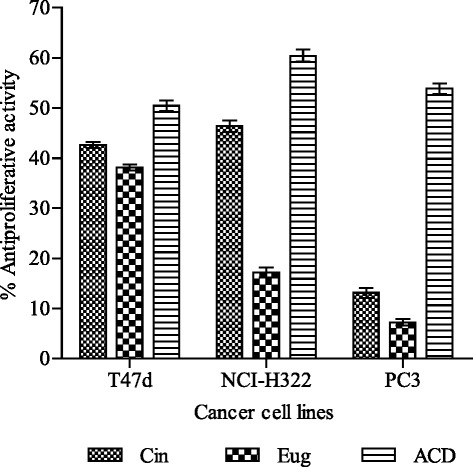
Antiproliferative activity of cinnamaldehyde and eugenol against cancer cell lines. Results are expressed as % inhibition on growth of cell lines (mean ± SD, *n* = 3). Abbreviations: *T47D* breast, *NCI-H322* lung, *PC-3* prostate cancer cell lines, *Cin* cinnamaldehyde, *Eug* eugenol, *ACD* anticancer drugs [Mitomycin (1 μM) against breast and prostate cancer cell lines; 5-fluro uracil (5 μM) against lung cancer cell line]

Moderate antiproliferative activity (43–46 %) was observed with cinnamaldehyde against breast (T47D) and lung (NCI-H322) cancer cell lines at 50 μM concentration while very low activity was observed against prostate (PC-3) cancer cell line at the same concentration in SRB assay (Fig. 6). In comparison to cinnamaldehyde, eugenol exhibited lower activity viz., 39, 17 and 13 % against T47D, NCIH-322 and PC-3 cell lines, respectively. Standard anticancer drugs mitomycin (1 μM) used against breast and prostate cancer cell lines and 5-Flurouracil (5 μM) used against lung cancer cell line showed 50–60 % antiproliferative activity only.

## Discussion

Plants are natural repositories of molecules with diverse structural and functional attributes. Many phytoconstituents exhibit nutritive and pharmacological activities [23–25]. They interact with different molecular and cellular targets including enzymes, hormones, trans-membrane transporters, and neurotransmitter receptors [26, 27]. Number of plant species and their metabolites have been identified and studied for their use in the pharmaceutical, medical, and agricultural industries [28]. Current work reports the biological activities of cinnamaldehyde and eugenol, the two phenylpropanoids, which are abundantly available in cinnamon and clove oils. Cinnamaldehyde occurs naturally in the bark of *Cinnamomum zeylanicum*, *C. cassia* and *C. camphora*. Essential oil of cinnamon bark contains about 80 % cinnamaldehyde and 10 % eugenol while cinnamon leaf oil contains 5 % cinnamaldehyde and about 95 % eugenol [29]. Eugenol is also present in essential oil fractions of *S. aromaticum*, *Myristica fragrans* and *Ocimum basilicum*. About 80–90 % eugenol is present in essential oils obtained from clove bud and leaf [30].

Free radicals play important role in the development of many chronic diseases including heart disease, cancer and the aging process [31]. To counteract their adverse effects‚ scavenging or reducing the formation of free radicals in the body becomes significant for health. In the present study cinnamaldehyde and eugenol have shown capability to scavenge free radical in DPPH radical scavenging assay (Fig. 2). DPPH is a stable nitrogen centered free radical and its color changes from violet to yellow upon uptake of hydrogen or electrons [32]. The effect of cinnamaldehyde and eugenol on DPPH is thought to be due to their hydrogen donating ability. Eugenol demonstrated greater radical scavenging activity as compared to cinnamaldehyde because it easily donates hydrogen atom of hydroxyl (OH) moiety directly linked to benzene ring [33]. Radical scavenging activity of the compounds depends on number and position of hydroxyl groups on aromatic ring of phenolic compounds and therefore they show ability to reduce the free radical level [25, 34]. Radical scavenging action of eugenol is further supported by the work of Mathew and Emilia [35]. They reported that eugenol exhibited a faster reaction rate and stronger intensities of white-yellow spots on thin layer chromatography (TLC) plates as compared to cinnamaldehyde and cinnamon bark extract [35]. Our results have shown better radical scavenging activity of eugenol (IC_50_ value 0.495 μM/ml) as compared with the report of Tominaga et al. [36].

Reducing power of bioactive compounds is often used as an indicator of electron donating activity, which is an important mechanism of antioxidant action [37]. Antioxidants can be reductants which inactivate oxidants. The reducing ability is measured by the direct reduction of ferricyanide (Fe^3+^) to ferrocyanide (Fe^2+^) which makes a Perl’s Prussian blue complex after addition of FeCl_3_ and absorbance is monitored at 700 nm. Increasing absorbance indicates an increase in reducing ability. The experimental data obtained in the current work indicated remarkable reducing potential in eugenol as compared to cinnamaldehyde (Fig. 3). Comparatively higher reducing power of eugenol might be due to the di and mono hydroxyl substitutions in the aromatic ring, which possess potent hydrogen or electron donating abilities [33, 38].

The process of lipid peroxidation, a free radical mediated chain reaction, is related to injury and inflammation and often associated with oxidative damage of membrane lipids [39]. It is usually initiated by hydroxyl radical which is produced through Fenton reaction in presence of metal ion (Fe^2+^) [40]. Lipid peroxidation may be enzymatic and non-enzymatic or both. Non enzymatic reaction involves three phases namely, initiation, propagation and termination [41]. Lipid, lipoperoxyl, lipid hydro-peroxide, peroxyl and alcoxyl radicals produced in the first two phases of lipid peroxidation are harmful to the body. Malondialdehyde (MDA) is an important product of lipid peroxidation which reacts with thiobarbituric acid (TBA) to form TBA-MDA adduct with an absorption maxima at 532 nm [42]. In the study cinnamaldehyde accounted for 33 % reduction in adduct formation at 3 μM/ml concentration signifying its lipoprotective action while lower activity was shown by eugenol (Fig. 4). Protective activity shown by test compounds could be attributed to the chelation of metal ion (Fe^3+^) which is responsible for generation of hydroxyl radical [43]. The antioxidants are believed to intercept the free radical chain of oxidation and donate hydrogen from the phenolic hydroxyl groups, thereby forming a stable end product that does not initiate or propagate further oxidation of lipid.

Transition metals catalyze the formation of the free radicals which initiate and propagate chain reaction in lipid peroxidation. Metal chelating capability indicates efficiency of a compound to protect lipids against oxidative damage [31]. Metal ions are quantitatively measured by ferrozine which make complexes with Fe^2+^ and gives pink colour. In the presence of chelating agents, the complex formation is disrupted with the result that the pink-red color of the complex is decreased. It has been reported that chelating agents act as a secondary antioxidants and form bonds with a metal because they reduce the redox potential, thereby stabilizing the oxidized form of the metal ion [44].

The present study reports that cinnamaldehyde has greater ion chelating ability as compared to eugenol (Fig. 5). This could be substantiated by the fact that aromatic aldehydes, especially with an effective conjugation system, form stable Schiff bases which are generally bi-, tri- or tetra- dentate chelate ligands and easily react with almost all transition metal ions and form very stable complexes with them. [45]. Moreover eugenol with the meta-methoxy groups and with the olefinic bond far away is not (well) suited for chelation (Fig. 1). This in contrast to that of cis-cinnamaldehyde, which forms chelates readily with low valent transition metals via pi-bonding of C = C and C = O bonds or via bonding of its C = C bond and the lone pair of C = O. Chelation reduces polarity of metal ion because of partial sharing of its positive charge with donor group in chelate ring system, The process of chelation thus increases the lipophilic nature of central ion. This in turn favours it permeation through the lipid layer of membrane which reduces hydroxyl radical generation at the site and thereby prevents initiation of lipid peroxidation [43]. Positive correlations between metal ion chelating ability and lipoprotective activity have also been reported earlier [46].

Emergence of multiple drug resistance in human pathogenic organisms has given momentum to search for new antimicrobial substances from alternative sources. Cinnamaldehyde exhibited considerable antibacterial activity against test bacteria (Table 1). There are reports showing appreciable killing potential in cinnamaldehyde against other bacteria [47, 48]. Comparatively lower activity was observed with eugenol. Cinnamaldehyde and eugenol are major ingredients of essential oils obtained from various species of genus *Cinnamomum*. A critical property of antibacterial components in essential oils is their hydrophobicity, which helps them to target the lipid-containing bacterial cell membrane and mitochondria [49]. In addition, these molecules can damage membrane proteins, deplete the proton motive force, cause leakage of cell contents and coagulate cytoplasm [50, 51]. Although these mechanisms might act independently, some of them could be activated as a consequence of another, resulting in a multiplicity of mechanisms [49].

The study performed by Shang et al. on four cinnamaldehyde congeners having similar structures proved that a conjugated double bond and a long CH chain outside the ring is responsible for better antibacterial activity of cinnamaldehyde. However presence of the hydroxyl group has also been shown to improve the antibacterial activity [52]. Trans-cinnamaldehyde has been reported to possess antimicrobial activity toward a wide range of foodborne pathogens [53, 54]. Eugenol has been shown to produce antibacterial effect against *Salmonella typhi* by damaging cytoplasmic membrane and causes subsequent leakage of intracellular constituents [55].

Cancer chemotherapeutic agent can often provide prolongation of life, temporary relief from symptoms and occasionally complete remission. A successful anticancer drug should kill or incapacitate cancer cells by inducing apoptosis in cancer cell. Toxicity caused by chemopreventive agents imposes restriction on their frequent usage and patients seek alternative methods of treatment. Hence there is need for developing new approaches and drugs from natural sources to treat cancer. Many important anticancer drugs are derived from plant sources such as taxol and camptothecin [38]. The free radical, especially hydroxyl radical, has ability to add to double bonds of DNA bases. It abstracts an H-atom from the methyl group of thymine and each of the five carbon atoms of deoxyribose at a very high rate constant resulting in permanent modification of genetic material leading to malfunctions of cellular process. Thus free radicals represent the first step involved in carcinogenesis [56]. Hence radical scavenging action shown by cinnamaldehyde and eugenol might play role in inhibiting initiation of carcinogenesis.

In the study cinnamaldehyde and eugenol displayed moderate antiproliferative activity against breast (T47D) and lung (NCI-H322) cancer cell lines (Fig. 6) showing anticancer potential which signifies their role in inhibiting cancer progression. Drugs being used for the cancer treatment follow different mechanisms of action. A number of herbal products have been reported to induce cell cycle arrest and thereby play important role in cancer prevention and therapy [57]. Furthermore, cinnamaldehyde has also been shown to inhibit cyclin dependent kinases (CDKs) which are involved in cell cycle regulation [58]. Many workers have reported cytotoxic effect of eugenol against human osteoblast (U2OS), fibroblast (HFF) and hepatoma (HepG2) cell lines [59, 60]. The modulation of angiogenesis, DNA (synthesis, transcription and translation), enzyme activity and microtubule inhibition remains an important therapeutic strategy against numerous diseases, including cancer [61]. Hence further studies are required for understanding the mechanism of action of cinnamaldehyde and eugenol as pharmacological agents.

## Conclusion

Cinnamaldehyde and eugenol exhibited considerable antioxidants, antimicrobials and moderate cytotoxic activities. Cinnamaldehyde showed lipo-protective and metal ion chelating abilities while eugenol accounted for radical scavenging and reducing activities. Cinnamaldehyde demonstrated appreciable antibacterial activity as compared to eugenol. The study will provide insight to researchers for utilization of these compounds for food and medicinal applications.

## Abbreviations

ACD, anticancer drugs; Asc, ascorbic acid; BHA, butylated hydroxyanisole; BHT, butylated hydroxytoluene; Cin, cinnamaldehyde; DPPH- 2, 2-Diphenyl-1-picryl hydrazyl; EDTA, ethylenediaminetetraacetic acid; Eug, eugenol; LPOI, lipid peroxidation inhibition; MTCC, microbial type culture collections; ROS, reactive oxygen species; SRB, sulforhodamine B dye

## References

[1] Maxwell SR (1995). Prospects for the use of antioxidant therapies. Drugs.

[2] Lopaczynski W, Zeisel SH (2001). Antioxidants, programmed cell death, and cancer. Nutrition Res.

[3] Halliwell B (1994). Free radicals, antioxidants and human disease: curiosity, cause, or consequence. Lancet.

[4] Ames BN, Shigenaga MK, Hagen TM (1993). Oxidants, antioxidants and the degenerative diseases of aging. Proc Natl Acad Sci U S A.

[5] Barcellos-Hoff MH (2005). Integrative radiation carcinogenesis interactions between cell and tissue responses to DNA damage. Semin Cancer Biol.

[6] Moukette BM, Pieme CA, Njimou JR, Biapa CPN, Marco B, Ngogang JY (2015). In vitro antioxidant properties, free radicals scavenging activities of extracts and polyphenol composition of a non-timber forest product used as spice: *Monodora myristica*. Biol Res.

[7] Pandey AK, Mishra AK, Mishra A, Kumar S, Chandra A (2010). Therapeutic potential of *C. zeylanicum* extracts: an antifungal and antioxidant perspective. Int J Biol Med Res.

[8] Kahl R, Kappus H (1993). Toxicity of synthetic antioxidants BHT and BHA in comparison with natural antioxidants vitamin E. Z Lebensm Unters Forsch.

[9] Mishra A, Kumar S, Bhargava A, Sharma B, Pandey AK (2011). Studies on in vitro antioxidant and antistaphylococcal activities of some important medicinal plants. Cell Mol Biol.

[10] Walker SJ (1988). Major spoilage microorganisms in milk and dairy products. J Soc Dairy Technol.

[11] Brewer MS, Sprouls GK, Russon C (1994). Consumer attitudes toward food safety issues. J Food Safety.

[12] Tripoli E, Guardia ML, Giammanco S, Majo DD, Giammanco M (2007). Citrus flavonoids: molecular structure, biological activity and nutritional properties: a review. Food Chem.

[13] Mishra A, Kumar S, Pandey AK (2013). Scientific validation of the medicinal efficacy of *Tinospora cordifolia*. Sci World J.

[14] Mishra AK, Mishra A, Kehri HK, Sharma B, Pandey AK (2009). Inhibitory activity of Indian spice plant *Cinnamomum zeylanicum* extracts against *Alternaria solani* and *Curvularia lunata*, the pathogenic dematiaceous moulds. Ann Clin Microbiol Antimicrob.

[15] Pandey AK, Mishra AK, Mishra A (2012). Antifungal and antioxidative potential of oil and extracts derived from leaves of Indian spice plant *Cinnamomum tamala*. Cell Mol Biol.

[16] Jaganathan SK, Supriyanto E (2012). Antiproliferative and molecular mechanism of eugenol-induced apoptosis in cancer cells. Molecules.

[17] Singh RP, Chidambara Murthy KN, Jayapakash GK (2002). Studies on the antioxidant activity of pomegranate *(Punica granatum*) peel and seed extracts using in vitro models. J Agric Food Chem.

[18] Oyaizu M (1986). Studies on products of browning reactions: anti oxidative activities of products of browning reaction prepared from glucosamine. Jap J Nutr.

[19] Halliwell B, Gutteridge JNC (1999). Mechanism of damage of cellular targets by oxidative stress: lipid peroxidation. Free Radicals in Biology and Medicine.

[20] Dinis TCP, Madeira VMC, Almeida LM (1994). Action of phenolic derivates (acetoaminophen, salicylate and 5-aminosalycilate) as inhibitors of membrane lipid peroxidation and as peroxyl radical scavengers. Arch Biochem Biophys.

[21] Bauer AW, Kirby WM, Sherris JC, Turck M (1966). Antibiotic susceptibility testing by a standardized single disk method. Am J Clin Pathol.

[22] Skehan P, Storeng R, Scudiero D, Monks A, McMahon J (1990). New colorimetric cytotoxicity assay for anticancer drug screening. J Natl Cancer Inst.

[23] Mishra AK, Mishra A, Bhargava A, Pandey AK (2008). Antimicrobial activity of essential oils from the leaves of *Cinnamomum* spp. Natl Acad Sci Lett.

[24] Mishra AK, Singh BK, Pandey AK (2010). In vitro-antibacterial activity and phytochemical profiles of *Cinnamomum tamala* (Tejpat) leaf extracts and oil. Rev Infect.

[25] Kumar S, Pandey AK (2013). Chemistry and biological activities of flavonoids: an overview. Sci World J.

[26] Harborne JB (2001). Twenty-five years of chemical ecology. Nat Prod Rep.

[27] Wink M, Acamovic T, Stewart CS, Pennycott TW (2004). Evolution of toxins and anti-nutritional factors in plants with special emphasis on Leguminosae. Poisonous plants and related toxins.

[28] Wink M (1993). The plant vacuole - a multifunctional compartment. J Exp Bot.

[29] Rao PV, Gan SH (2014). Cinnamon: a multifaceted medicinal plant. Evid Based Comp Altern Med.

[30] Barnes J, Anderson LA, Phillipson JD (2007). Herbal medicines.

[31] Kumar S, Gupta A, Pandey AK (2013). *Calotropis procera* root extract has the capability to combat free radical mediated damage. ISRN Pharmacol.

[32] Kumar S, Pandey AK (2014). Medicinal attributes of *Solanum xanthocarpum* fruit consumed by several tribal communities as food: an in vitro antioxidant, anticancer and anti HIV perspective. BMC Complement Altern Med.

[33] Gulcin I (2011). Antioxidant activity of eugenol: a structure-activity relationship study. J Med Food.

[34] Brand-Williams W, Cuvelier ME, Berset C (1995). Use of a free radical method to evaluate antioxidant activity. LWT-Food Sci Technol.

[35] Mathew S, Emilia AT (2006). Studies on the antioxidant activities of cinnamon (*Cinnamomum verum*) bark extracts, through various in vitro models. Food Chem.

[36] Tominaga H, Kobayashi Y, Goto T, Kasemura K, Nomura M (2005). DPPH radical-scavenging effect of several phenylpropanoid compounds and their glycoside derivatives. Yakugaku Zasshi.

[37] Gulcin I, Oktay M, Kirecci E, Kufrevioglu OI (2003). Screening of antioxidant and antimicrobial activities of anise (*Pimpinella anisum* L.) seed extracts. Food Chem.

[38] Shimada K, Fujikawa K, Yahara K, Nakamura T (1992). Antioxidative properties of xanthan on the autooxidation of soybean oil in cyclodextrin emulsion. J Agric Food Chem.

[39] Sharma AK, Kumar S, Pandey AK (2014). Ferric reducing, anti-radical and cytotoxic activities of *Tinospora cordifolia* stem extracts. Biochem Anal Biochem.

[40] Ganz T (2003). Hepcidin, a key regulator of iron metabolism and mediator of anemia of inflammation. Blood.

[41] Guéraud F, Atalay M, Bresgen N, Cipak A, Eckl PM (2010). Chemistry and biochemistry of lipid peroxidation products. Free Radic Res.

[42] Kumar S, Mishra A, Pandey AK (2013). Antioxidant mediated protective effect of *Parthenium hysterophorus* against oxidative damage using in vitro models. BMC Complement Altern Med.

[43] Shreaz S, Rayees A, Rimple SB, Hashmi AA, Nikhat M, Khan LA (2010). Anticandidal activity of cinnamaldehyde, its ligand and Ni(II) complex: effect of increase in ring and side chain. Microb Pathog.

[44] Gordon MH, Hudson BJF (1990). The mechanism of the antioxidant action in vitro. Food antioxidants.

[45] Adabiardakani A, Mohammad HHK (2012). Cinnamaldehyde schiff base derivatives: a short review. World Appl Program.

[46] Kumar S, Kumar R, Dwivedi A, Pandey AK (2014). In vitro antioxidant, antibacterial, and cytotoxic activity and in vivo effect of *Syngonium podophyllum* and *Eichhornia crassipes* leaf extracts on isoniazid induced oxidative stress and hepatic markers. BioMed Res Int.

[47] McCann J (2003). Herbal medicine handbook.

[48] Ates DA, Erdogrul OT (2003). Antimicrobial activities of various medicinal and commercial plant extracts. Turk J Biol.

[49] Burt S (2004). Essential oils: their antibacterial properties and potential applications in food- a review. Int J Food Microbiol.

[50] Ultee A, Kets EPW, Smid EJ (1999). Mechanisms of action of carvacrol on the food-borne pathogen *Bacillus cereus*. Appl Environ Microbiol.

[51] Sikkema J, De Bont JAM, Poolman B (1994). Interactions of cyclic hydrocarbons with biological membranes. J Biol Chem.

[52] Chang ST, Chen PF, Chang SC (2001). Antibacterial activity of leaf essential oils and their constituents from *Cinnamomum osmophloeum*. J Ethnopharmacol.

[53] Friedman M, Henik PR, Mandrell RE (2002). Bactericidal activities of plant essential oils and some of their isolated constituents against *Campylobacter jejuni*, *Escherichia coli*, *Listeria monocytogenes*, and *Salmonella enterica*. J Food Prot.

[54] Baskaran SA, Kazmer GW, Hinckley L, Andrew SM, Venkitanarayanan K (2009). Antibacterial effect of plant-derived antimicrobials on major bacterial mastitis pathogens in vitro. J Dairy Sci.

[55] Devi KP, Arif NS, Sakthivel R, Karutha PS (2010). Eugenol (an essential oil of clove) acts as an antibacterial agent against *Salmonella typhi* by disrupting the cellular membrane. J Ethnopharmacol.

[56] Dizdaroglu M, Jaruga P, Birincioglu M, Rodriguez H (2002). Free radical-induced damage to DNA: mechanisms and measurement. Free Radic Biol Med.

[57] Sharma AK, Kumar S, Chashoo G, Saxena AK, Pandey AK (2014). Cell cycle inhibitory activity of *Piper longum* against A549 cell line and its protective effect against metal-induced toxicity in rats. Ind J Biochem Biophys.

[58] Jeong HW, Kim MR, Son KH, Han MY, Ha JH, Garnier M, Meijer L, Kwon BM (2000). Cinnamaldehydes inhibit cyclin dependent kinase 4/cyclin D1. Bioorg Med Chem Lett.

[59] Ho YC, Huang FM, Chang YC (2006). Mechanisms of cytotoxicity of eugenol in human osteoblastic cells in vitro. Int Endodontic J.

[60] Babich H, Stern A, Borenfreund E (1993). Eugenol cytotoxicity evaluated with continuous cell lines. Toxicol In Vitro.

[61] Kumar S, Ahmad MK, Waseem M, Pandey AK (2015). Drug targets for cancer treatment: an overview. Med Chem.

